# Non-alcoholic fatty liver disease and gestational diabetes mellitus: a bidirectional two-sample mendelian randomization study

**DOI:** 10.1186/s12902-024-01569-6

**Published:** 2024-03-20

**Authors:** Ben-Gang Zhou, Jian-Lei Xia, Xin Jiang, Yan-Bing Ding, Qiang She

**Affiliations:** 1 Dalian Medical University, Dalian, Liaoning Province China; 2 Department of Gastroenterology, Affiliated Hospital of Yangzhou University, Yangzhou University, No. 368, Hanjiang Middle Road, Hanjiang District, Yangzhou, Jiangsu Province China

**Keywords:** Nonalcoholic fatty liver disease, Gestational diabetes mellitus, Mendelian randomization, Causality

## Abstract

**Purpose:**

Previous observational studies have revealed a potential link between non-alcoholic fatty liver disease (NAFLD) and gestational diabetes mellitus (GDM), but their causal relationship remains unclear. Thus, this study aimed to examine whether a causal link exists between genetically determined NAFLD and GDM.

**Methods:**

Utilizing publicly accessible genome-wide association studies (GWAS), a two-sample bidirectional Mendelian randomization (MR) analysis was conducted. The GWASs data pertaining to NAFLD and GDM were obtained from the UK Biobank Consortium and FinnGen database in primary analysis, respectively. The random-effects inverse variance weighted (IVW) method was utilized as primary analysis method. Several sensitivity analyses were utilized to verify the robustness of the results. Additionally, we also analyzed the causal effect of potential shared influencing factors on these two conditions.

**Results:**

The result of the IVW method showed that there was no significant causal relationship between genetically determined NAFLD and GDM (OR = 0.98, 95% CI: 0.90–1.07, *P* = 0.691). Similarly, our reverse MR analysis failed to detect a significant causal effect of GDM on NAFLD (OR = 1.14, 95% CI: 0.97–1.36, *P* = 0.118). Sensitivity analyses further confirmed the robustness of the results. Moreover, we found that genetically determined body mass index, waist-to-hip ratio, triglycerides, and television viewing time may be positively correlated with NAFLD and GDM, while high-density lipoprotein cholesterol and apolipoprotein A-I may both be negatively correlated with NAFLD and GDM.

**Conclusions:**

The current bidirectional MR study failed to provide sufficient genetic evidence for the causal relationship between NAFLD and GDM.

**Supplementary Information:**

The online version contains supplementary material available at 10.1186/s12902-024-01569-6.

## Introduction

Non-alcoholic fatty liver disease (NAFLD) is a common disorder that is characterized by an excessive amount of fat stored in the liver of people who do not consume large amounts of alcohol or have other liver diseases [1]. NAFLD has become the most common chronic liver disease worldwide, with an estimated prevalence rate of 30% among adults globally [2]. A recent meta-analysis involving over 1.2 million people showed that the incidence rate of NAFLD was 46.13 per 1000 person years, with considerable disparities in gender, body mass index (BMI), geography and time-period [3]. An increasing amount of evidence indicates that NAFLD is a multisystem disease, and its clinical and economic impacts are not limited to the progression of liver disease (nonalcoholic steatohepatitis, liver fibrosis, cirrhosis and hepatocellular carcinoma) but are also linked to an increased risk of numerous extrahepatic diseases [4–7].

Gestational diabetes mellitus (GDM) is the most common medical complication in pregnant women, referring to any glucose intolerance that is identified or develops during pregnancy [8, 9]. It has been observed that the prevalence of GDM varies greatly across the world, with some countries having a rate of 1% while others have a rate of over 30% [9].

GDM put pregnant women and their newborns at risk in several ways, including higher chances of adverse pregnancy outcomes like hypertension, pre-eclampsia, preterm delivery, macrosomia, respiratory distress syndrome and neonatal jaundice. Furthermore, it can also have a lasting effect on both mother and child, including type 2 diabetes, metabolic syndrome, cardiovascular and cerebrovascular diseases [9–11]. Nevertheless, the cause of GDM is yet to be determined.

In the last few years, the association between NAFLD and GDM has been a subject of great fascination for researchers. Previous observational studies showed that NAFLD was associated with GDM in this rural south Asian community [12]. Several observational studies also indicated that GDM were at increased risk of developing NAFLD [13, 14]. A recent cohort study of Korean adults showed that a history of GDM was an independent risk factor for the emergence of NAFLD. The correlation between GDM and NAFLD events was only explained by insulin resistance (IR) measured by homeostasis model assessment of insulin resistance (HOMA-IR) and the development of diabetes to a limited extent (10%) [15]. However, correlation does not equate to causation; it merely reflects the statistical connection between two variables that can be measured [16]. To date, the causal relationship between NAFLD and GDM has not yet been fully established.

By employing genetic variants as instrumental variables (IVs), Mendelian randomization (MR) is an analytical approach that can better ascertain the causality of exposure towards an outcome [17]. Unlike observational studies, MR analyses are not influenced by common confounding factors like postnatal environment, socioeconomic status and behavioural factors, as alleles are randomly and independently segregated during meiosis [17, 18]. Moreover, since genetic variations are fixed from birth and remain the same throughout life, MR can help prevent reverse causality bias [19].

Hence, this study aims to investigate the causal relationship between NAFLD and GDM through a bidirectional MR analysis of two samples to examine whether NAFLD is a cause of GDM and if GDM is a risk factor for NAFLD. Given the high prevalence and burden of NAFLD and GDM, we believe that elucidating the causal relationship between NAFLD and GDM is of great clinical significance for the development of future prevention and treatment strategies for both conditions.

## Methods

### Ethical statement and reporting guidance

As this study was based on a re-analysis of previously conducted and published genome-wide association studies (GWASs) data, there is no requirement for further ethical approval. All original studies were conducted with the necessary ethical approval and participant consent. This study was conducted following the Strengthening the Reporting of Observational Studies in Epidemiology using MR (STROBE-MR) guideline [20].

### Study design

**Fig. 1:**
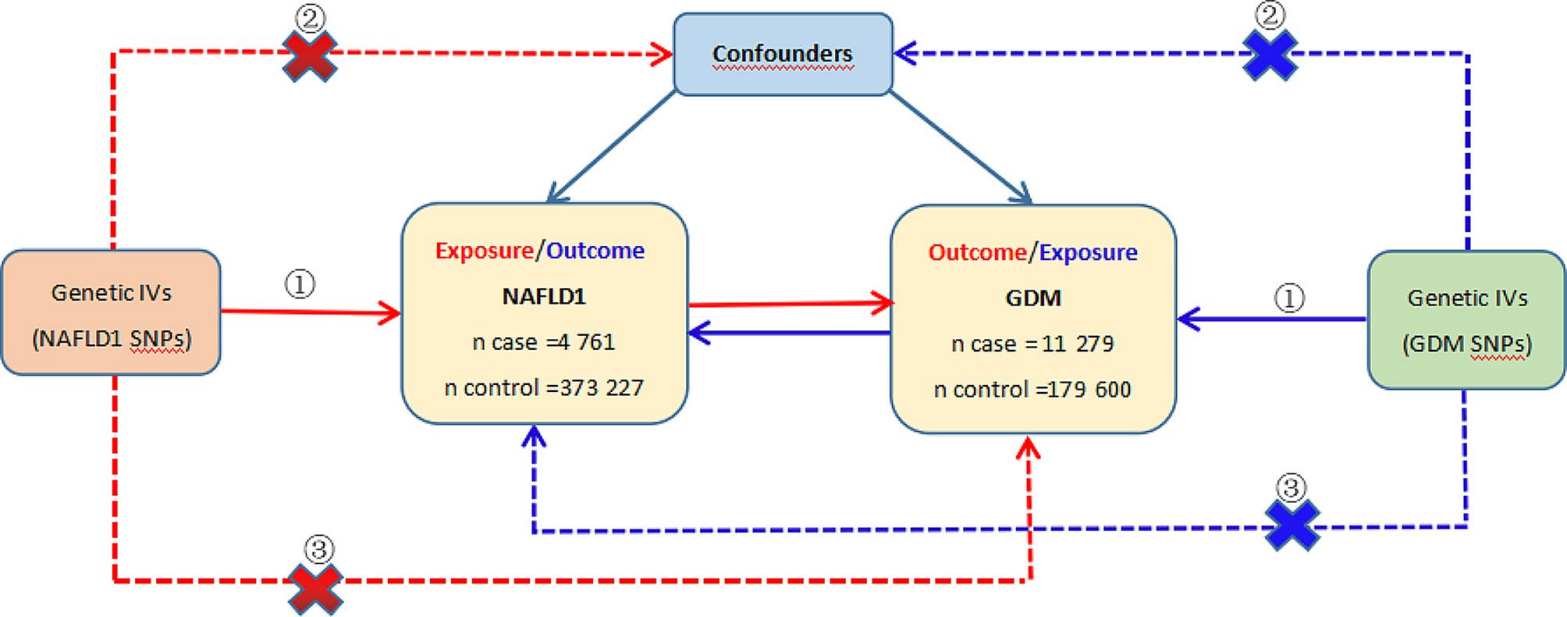
The flow chart of bidirectional primary MR analyses. IVs, instrumental variants; SNP, single-nucleotide polymorphism. NAFLD, non-alcoholic fatty liver disease; GDM, gestational diabetes mellitus; ① relevance assumption; ② independence assumption; ③ exclusion-restriction assumption

**Fig. 2:**
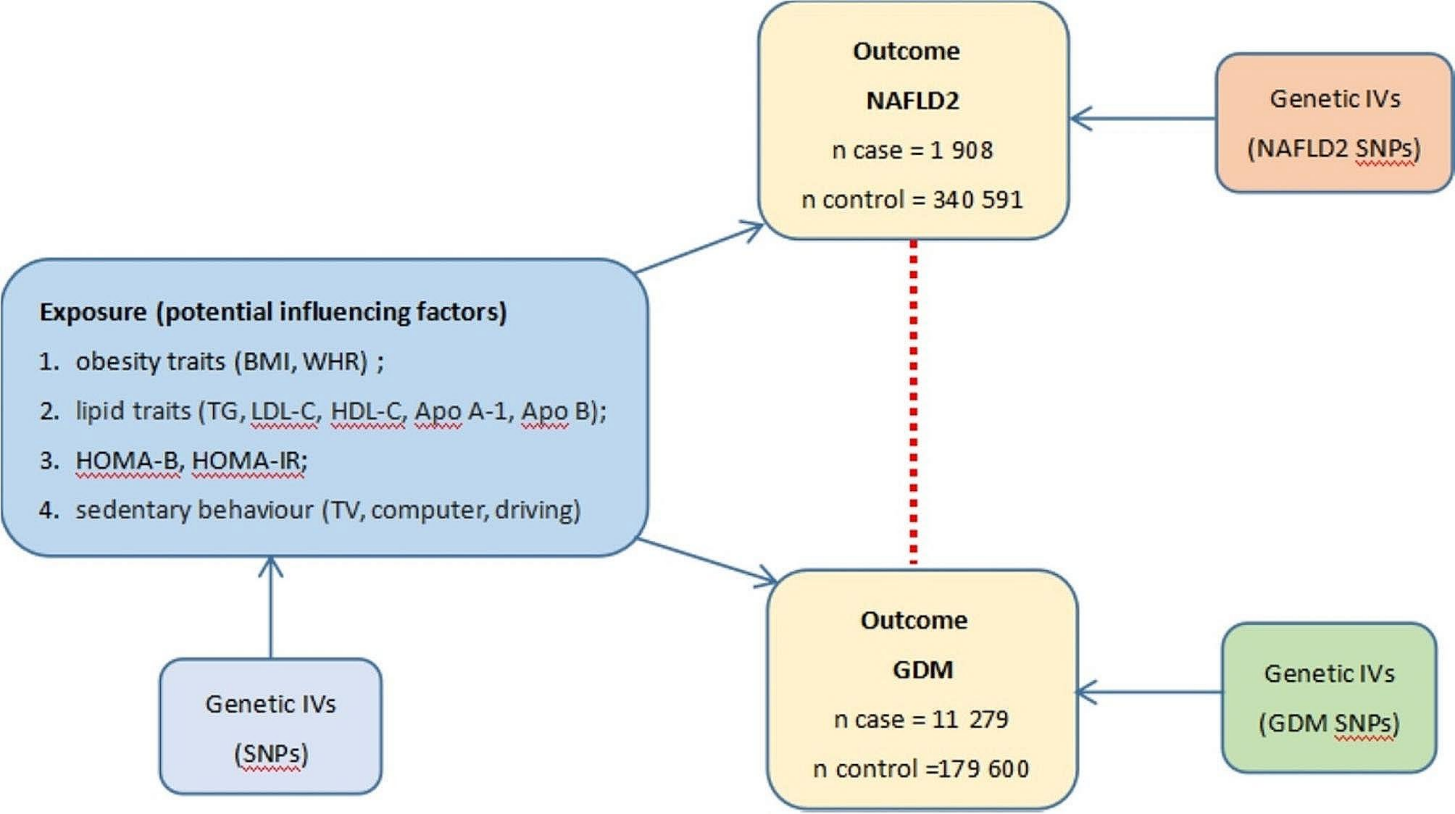
The flow chart of the causal effect of potential shared influencing factors on NAFLD or GDM in the secondary MR analysis. IVs, instrumental variants; SNP, single-nucleotide polymorphism. NAFLD, non-alcoholic fatty liver disease; GDM, gestational diabetes mellitus; BMI, body mass index; WHR, waist-to-hip ratio; TG, triglycerides; LDL-C, low-density lipoprotein cholesterol; HDL-C, high-density lipoprotein cholesterol; Apo A-1, apolipoprotein A-I; Apo B, apolipoprotein B; HOMA-B, homeostasis model assessment of beta-cell function; HOMA-IR, homeostasis model assessment of insulin resistance; TV, Time spent watching television; Computer, Time spent using computer; Driving, Time spent driving

A two-sample bidirectional MR was conducted to assess the causal relationship between NAFLD and GDM. The MR study was conducted based on three essential assumptions: (1) the relevance hypothesis, which suggests that genetic variation is closely associated with exposure; (2) the independence hypothesis, which asserts that genetic variation is not linked to any confounding factors that could mediate between exposure and outcome; and (3) the exclusion restriction hypothesis, which establishes that genetic variation can solely influence outcomes through exposure [18]. The first step was to investigate the causal effect of NAFLD on GDM. Then, the second step to explore the causal influence of GDM on NAFLD. The overall flow chart of primary MR analysis was presented in Fig. 1. Furthermore, to explore whether there were intermediate factors influencing the causal relationship between NAFLD and GDM, we further analyzed the causal effect of potential shared influencing factors on these two conditions. These influencing factors included obesity traits (BMI and waist-to-hip ratio [WHR]), lipid traits (triglycerides [TG], low-density lipoprotein cholesterol [LDL-C], high-density lipoprotein cholesterol [HDL-C], apolipoprotein A-I [Apo A-1], and apolipoprotein B [Apo B]), homeostasis model assessment of beta-cell function (HOMA-B), HOMA-IR, and sedentary behaviour (time spent watching television [TV], time spent using computer [Computer], and time spent driving [Driving]). Figure 2 shows the causal effects of potential shared influencing factors on NAFLD or GDM.

### GWASs data sources

The data for GWASs pertaining to GDM was obtained from the FinnGen database (https://r8.finngen.fi/), which comprises 190 897 participants (11 279 cases and 179 600 controls) of European ancestry. The genetic IVs of NAFLD were sourced from two separate sets of GWAS data. One dataset (NAFLD1) from the UK Biobank Consortium (http://www.nealelab.is/uk-biobank) was utilized to conduct primary MR analysis, which was recently published by Fairfield et al. [21] The other dataset (NAFLD2) from the FinnGen database (https://r8.finngen.fi/) was used to perform secondary MR analysis. In addition, the GWASs data for obesity traits (BMI and WHR), lipid traits (TG, LDL-C, HDL-C, Apo A-1, Apo B), HOMA-B (IR), and sedentary behaviour (TV, Computer, Driving) were derived from Genetic Investigation of Anthropometric Traits (GIANT) consortium (https://portals.broadinstitute.org/collaboration/giant/index.php/GIANT_consortium), the UK Biobank Consortium (http://www.nealelab.is/uk-biobank), the Meta-analyses of Glucose and Insulin-Related Traits Consortium (MAGIC) (http://magicinvestigators.org/downloads/), and the Medical Research Council Integrative Epidemiology Unit (MRC-IEU), respectively. Table 1 provides an overview of the GWASs data that were used in this study.

**Table 1:** Details of the GWASs data included in the Mendelian randomization analyses

Phenotypes	Consortium	Ethnicity	Sample size	Number of SNPs	IEU GWAS ID	PMID
GDM	FinnGen	European	190,897	20,160,256	NA	NA
NAFLD1	UK Biobank	European	377,988	9,211,209	NA	34,535,985
NAFLD2	FinnGen	European	342,499	20,169,557	NA	NA
BMI	GIANT	European	681,275	2,336,260	ieu-b-40	30,124,842
WHR	GIANT	European	212,244	2,560,782	ieu-a-73	25,673,412
TG	UK Biobank	European	441,016	12,321,875	ieu-b-111	32,203,549
LDL-C	UK Biobank	European	440,546	12,321,875	ieu-b-110	32,203,549
HDL-C	UK Biobank	European	403,943	12,321,875	ieu-b-109	32,203,549
Apo A-1	UK Biobank	European	393,193	12,321,875	ieu-b-107	32,203,549
Apo B	UK Biobank	European	439,214	12,321,875	ieu-b-108	32,203,549
HOMA-B	MAGIC	European	36,466	2,454,220	ieu-b-117	20,081,858
HOMA-IR	MAGIC	European	37,037	2,455,342	ieu-b-118	20,081,858
TV	MRC-IEU	European	437,887	9,851,867	ukb-b-5192	NA
Computer	MRC-IEU	European	360,895	9,851,867	ukb-b-4522	NA
Driving	MRC-IEU	European	310,555	9,851,867	ukb-b-3793	NA

Abbreviations: GDM, gestational diabetes mellitus; NAFLD, nonalcoholic fatty liver disease; BMI, body mass index; WHR, waist-to-hip ratio; TG, triglycerides; LDL-C, low-density lipoprotein cholesterol; HDL-C, high-density lipoprotein cholesterol; Apo A-1, apolipoprotein A-I; Apo B, apolipoprotein B; HOMA-B, homeostasis model assessment of beta-cell function; HOMA-IR, homeostasis model assessment of insulin resistance; TV, Time spent watching television; Computer, Time spent using computer; Driving, Time spent driving; GIANT, Genetic Investigation of ANthropometric Traits; MAGIC, Meta-Analyses of Glucose and Insulin-related traits Consortium; MRC-IEU, Medical Research Council Intergrative Epidemiology Unit; SNPs, single-nucleotide polymorphisms; PMID, ID of publication in the PubMed; NA, not applicable

### Genetic instrument selection

The selection of genetic IVs was based on the following criteria: (1) we screened the IVs using a genome-wide significance threshold (*P* < 5 × 10^− 8^) in all phenotypes, except for HOMA-B and HOMA-IR, where we set a more relax threshold (*P* < 5 × 10^− 6^) to obtain more SNPs; (2) we removed single-nucleotide polymorphisms (SNPs) that could potentially have linkage disequilibrium (r ^2^ < 0.001 at a 10,000-kilobase window); (3) we excluded palindromic SNPs, and SNPs related to the outcomes, whose *P* value was below the nominal *P* value Bonferroni-corrected for the number of SNPs; (4) we made sure that all independent variables had an F statistics exceeding 10 and eliminated SNPs with F values below 10 to mitigate the risk of weak instrument bias. To calculate the F statistics, we used the following formula: F = (β_exposure_ × β_exposure_)/(SE _exposure_ × SE _exposure_) [22].

### Statistical analysis

The primary statistical analysis method utilized was the random-effects inverse variance weighted (IVW) method, which was complemented by three sensitivity analyses: the weighted median method (WM), MR-Egger, and MR pleiotropy residual sum and outlier (MR-PRESSO). By constraining the intercept to zero, the point estimates yielded by IVW MR can be viewed as a weighted linear regression of SNP-outcome associations against SNP-exposure associations [23]. The IVW method provides the most accurate estimation based on the assumption of no imbalanced horizontal pleiotropy [24]. The WM method was defined as the median of a weighted empirical density function of the ratio estimates. If at least 50% of the selected SNPs are valid, the WM estimator may yield unbiased causal effects [23, 25]. The MR-Egger method was utilized to check horizontal pleiotropy, providing accurate estimates even when all the SNPs are invalid in an instrument. Horizontal pleiotropy was indicated by the MR-Egger intercept with a *P*-value lower than 0.05 [26, 27]. To address horizontal pleiotropic outliers in multi-instrument summary-level MR testing, the MR-PRESSO approach was utilized for identification and correction. This approach can detect outliers and provide estimates after the removal of outliers [28]. Cochran’s Q test and I^2^ statistic were conducted to evaluate the heterogeneity between different genetic IVs. I^2^ > 50% indicated significant heterogeneity [29]. Additionally, the leave-one-out analysis was carried out to assess the stability of the results, that is, whether the MR estimate was influenced by a single SNP.

The outcomes are presented in the form of odds ratios (ORs) along with their corresponding 95% confidence intervals (CIs). A causal association was deemed statistically significant if the p-value was less than 0.05. In the secondary analysis, a Bonferroni correction was applied and an adjusted *P*-value of less than 0.004 (0.05/12) was considered a significant association. When the *P-*values were greater than 0.004 but less than 0.05, they were deemed as suggestive associations.

All statistical analyses were conducted using “TwoSampleMR” (https://github.com/MRCIEU/TwoSampleMR) and “MR-PRESSO”(https://github.com/rondolab/MR-PRESSO) packages in the R software (version 4.2.3).

## Results

### Causal effect of NAFLD on GDM

**Fig. 3:**
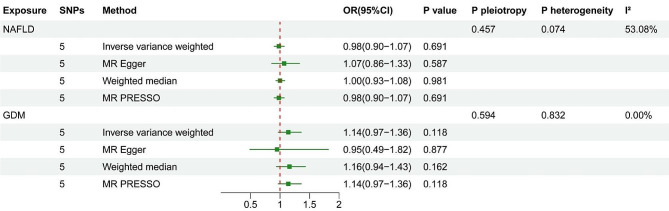
The results of bidirectional primary MR analyses

A total of 5 SNPs were selected in our MR analyses. The characteristics of NAFLD-related SNPs and F statistics were presented in Supplementary Table S1. The F statistic for each selected SNP was over 30, indicating that all selected SNPs had sufficient validity. As shown in Fig. 3, the result of the IVW method showed that there was no significant causal relationship between genetically determined NAFLD and GDM (OR = 0.98, 95% CI: 0.90–1.07, *P* = 0.691). Similar results were observed with MR-Egger method (OR = 1.07, 95% CI: 0.86–1.33, *P* = 0.587), WM method (OR = 1.00, 95% CI: 0.93–1.08, *P* = 0.981) and MR-PRESSO method (OR = 0.98, 95% CI: 0.90–1.07, *P* = 0.691). Significant heterogeneity was observed in the IVW analysis (I^2^ = 53.08%). The Egger intercept test did not reveal any indication of horizontal pleiotropy (*P* = 0.457). The overall MR estimate did not show any significant change upon removal of a single instrument SNP in the leave-one-out sensitivity analysis, which suggested the MR results were robust (Supplementary Figure S1). The forest plot and scatter plot were presented in Supplementary Figure S2 and Figure S3.

### Causal effect of GDM on NAFLD

The MR analysis focused on 5 GDM-related SNPs, each of which had an F-value greater than 10. The characteristics of these SNPs were summarized in Supplementary Table S2. As shown in Fig. 3, the IVW analysis conducted did not reveal any significant causal links between genetically determined GDM and NAFLD (OR = 1.14, 95% CI: 0.97–1.36, *P* = 0.118). The MR-Egger, WM and MR-PRESSO methods also supported the conclusion (Fig. 3). Neither horizontal pleiotropy (*P* = 0.594) nor heterogeneity (*P* = 0.832, I^2^ = 0%) was observed. Additionally, the leave-one-out analysis did not reveal any SNPs that significantly influenced the combined estimate (Supplementary Figure S4). The forest plot and scatter plot were presented in Supplementary Figure S5 and Figure S6.

### Causal effect of potential shared influencing factors on NAFLD or GDM

**Fig. 4:**
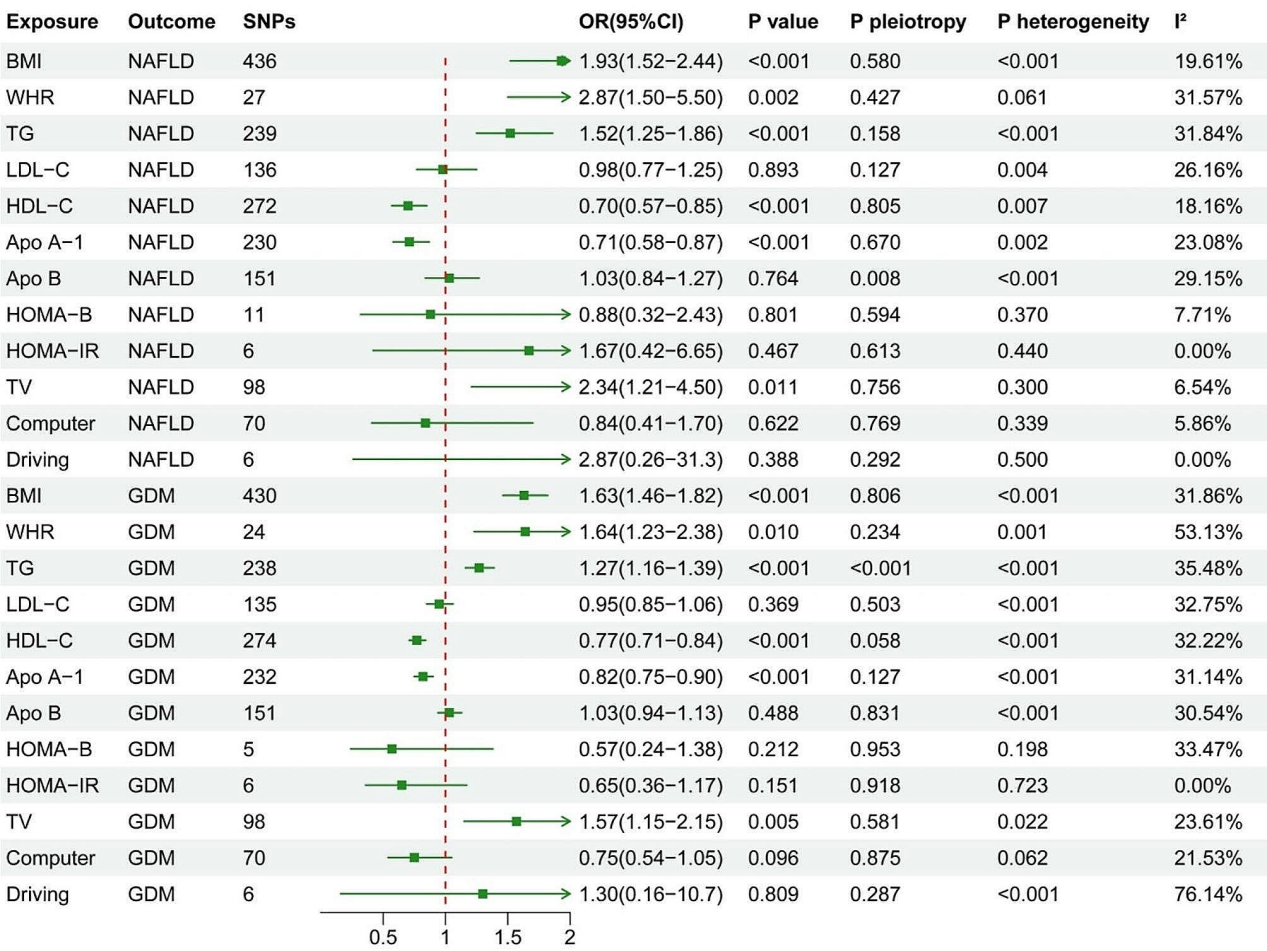
The results of causal effect of potential shared influencing factors on NAFLD or GDM

MR analyses were further conducted to explore the causal effect of potential influencing factors on NAFLD or GDM. These factors included obesity traits (BMI and WHR), lipid traits (TG, LDL-C, HDL-C, Apo A-1, Apo B), HOMA-B, HOMA-IR, and sedentary behaviour (TV, Computer, Driving). Figure 4 summarizes the results of IVW as the primary analysis. According to the IVW method, genetically determined BMI, WHR, TG, and time spent watching TV may be positively associated with NAFLD, while HDL-C and Apo A-1 may have a negative association with NAFLD. No causal effects were observed from LDL-C, Apo-B, HOMA-B, HOMA-IR, Computer, and Driving on NAFLD. There was no horizontal pleiotropy present except for Apo-B. No significant heterogeneity was observed.

With regard to the causal effect of potential shared influencing factors on GDM, similarly, we also found that genetically determined BMI, WHR, TG, and time spent watching TV was positively associated with GDM, while HDL-C and Apo A-1 may have a negative association with NAFLD in the IVW analysis. No causal effects were observed from LDL-C, Apo-B, HOMA-B, HOMA-IR, Computer, and Driving on GDM. There was no horizontal pleiotropy present except for TG. No significant heterogeneity was observed except for WHR. The results of IVW, MR-Egger, WM and MR-PREESO method were presented in Supplementary Table S3 and Table S4.

## Discussion

To the best of our knowledge, the MR study is the first to explore the potential bidirectional causal association between NAFLD and GDM. Currently, there is no sufficient genetic evidence to suggest that NAFLD causes GDM, or that GDM causes NAFLD. The MR causal effect estimates were confirmed to be robust and reliable through multiple sensitivity analyses.

So far, the relationship between NAFLD and GDM has not been confirmed. Previous observational studies or meta-analyses have indicated a mutual relationship between them. In 2011, Forbes et al. [30] conducted a cross-sectional study and showed that compared to European women without a history of GDM, ultrasound-diagnosed NAFLD was significantly more prevalent in those with a history of GDM. Nevertheless, the study did not consider pre-pregnancy metabolic risk factors and the sample size was inadequate. A multicentre prospective cohort study from pregnant Korean women showed that the risk of GDM was considerably higher in those with NAFLD and was linked to the severity of steatosis. This association between NAFLD and GDM kept its significance even after considering metabolic risk factors, including indicators of insulin resistance [31]. A recent population-based prospective cohort study from rural south Asian community also showed that NAFLD may be a major risk factor for GDM [12]. A recent meta-analysis suggested that NAFLD was associated with multiple pregnancy related diabetic complications [32]. In addition, research suggested that GDM may also be linked to an increased risk of developing NAFLD. A meta-analysis of three cohort studies indicated a significantly higher risk of developing NAFLD after a GDM diagnosis (OR = 2.60, 95% CI:1.90–3.57, I^2^ = 0%) [14]. Similar results were observed in a recent cohort study from Korean [15].

Despite the fact that our research evidence, based on MR analysis, failed to demonstrate a bidirectional causal relationship between NAFLD and GDM, several potential mechanisms could exist to explain the association between NAFLD and GDM. Firstly, NAFLD and GDM were two distinct metabolic illnesses, both of which could be linked to a shared metabolic abnormality such as IR [33, 34]. IR served as a key factor in the relationship between the development of GDM and NAFLD, although a recent study [15] indicated that its mediating impact on this connection was less than 10%. Secondly, the mechanism of how NAFLD leads to impaired glucose tolerance is likely due to a shared pro-inflammatory response of both adipokines and hepatokines [35]. Low levels of adiponectin and high levels of selenoprotein-P were linked to the sonographic and biochemical severity of NAFLD, as well as being independent predictors of late pregnancy GDM. These inflammatory markers could potentially serve as valuable biomarkers in the future for gauging the severity of NAFLD and the likelihood of developing GDM, regardless of other metabolic factors [31, 32]. Thirdly, women with a prior history of GDM, who have decreased insulin sensitivity and increased insulin secretion, may be more prone to developing NAFLD due to compensatory hyperinsulinemia, as insulin is known to stimulate hepatic lipogenesis. Insulin sensitivity impairment reduces the ability to suppress hepatic glucose production and insulin-stimulated glucose uptake in skeletal muscle, as well as increases fatty acid production from adipose tissue, ultimately resulting in a higher influx of fatty acids to the liver, consequently causing the emergence of NAFLD [30, 36–38]. Additionally, women with a history of GDM may have lower levels of adiponectin or other adipocytokines, which could be a factor in the pathophysiological pathways connecting GDM and NAFLD [39]. To sum up, the detailed mechanisms underlying the relationship between NAFLD and GDM are still not completely understood and require additional study in the future.

Considering that NAFLD and GDM may shared common risk factors, in order to explore whether there were intermediate factors influencing the causal relationship between NAFLD and GDM, we further analyzed the effects of obesity traits, lipid traits, HOMA-B, HOMA-IR, and sedentary behaviour on NAFLD or GDM. The MR analyses demonstrated that genetically determined BMI, WHR, TG, and time spent watching TV may be positively associated with both NAFLD and GDM, while HDL-C and Apo A-1 may have a negative association with both NAFLD and GDM. Considering that the previous observational studies did not fully adjust these possible influencing factors, these possible influencing factors may be one of the reasons why previous observational studies have found a correlation between NAFLD and GDM.

While our study concluded that there was no significant causal relationship between NAFLD and GDM based on the MR analysis, it is essential to acknowledge the limitations associated with null findings. Null results may be indicative of insufficient statistical power, genetic instrument validity issues, or complexities in the relationship between the exposures and outcomes, rather than definitive evidence of absence of causality. Given that no single source of evidence can establish a conclusive causal relationship, it is essential to interpret MR research results based on comprehensive evidence. This involves integrating the findings of various studies with different study designs to arrive at more reliable conclusions for a specific causal relationship issue [40].

### Strengths and limitations

Our MR study has several strengths. Firstly, as mentioned earlier in the introduction, in contrast to observational epidemiological studies, MR studies have a notable advantage in that they are less susceptible to bias from confounders or reverse causation. Secondly, the reliable evidence for the bidirectional relationship between NAFLD and GDM is supported by both the consistency of main effect estimation and sensitivity analysis of various methods. Thirdly, considering that the GWASs we relied on for our study only involved individuals of European ancestry and had genomic controls, it is improbable that our MR findings were influenced by population stratification.

Nevertheless, it is important to acknowledge several limitations that must be taken into account in this MR study. First, the GWAS dataset utilized in our MR analysis was obtained from European populations, which may limit the generalizability of the results to other ethnic or geographical populations. More studies are needed to determine whether a causal link exists between NAFLD and GDM in other regions. Second, since the data was limited, we were unable to stratify the analysis based on the severity of NAFLD. Third, despite using different techniques to remove outliers and variants with pleiotropy, and the horizontal pleiotropy test not indicating any problems, we cannot be certain that our results were not affected by unidentified pleiotropic variants.

### Future directions

Given the complexity of NAFLD and GDM etiology, our study could provide insights into future research directions, such as investigating gene-environment interactions or conducting larger prospective longitudinal studies to assess the temporal relationship between NAFLD and GDM. Furthermore, our research primarily focuses on the GWAS data of the European population. Once GWAS data from other populations becomes available, future studies should investigate the causal link between NAFLD and GDM in non-European populations. Additionally, if there is available data in the future, it is crucial to explore the causal relationship between the severity of NAFLD (such as non-alcoholic steatohepatitis, liver fibrosis, etc.) and GDM.

In conclusion, our current bidirectional MR study failed to provide sufficient genetic evidence for the causal relationship between NAFLD and GDM. Further updated MR analysis is necessary to confirm our findings once a comprehensive and more detailed GWAS database of NAFLD and GDM patients becomes accessible.

### Electronic supplementary material

Below is the link to the electronic supplementary material.

Supplementary Material 1

## Data Availability

Data is provided within the manuscript or supplementary information files. Publicly available data were analyzed in this study, which can be found here: https://r8.finngen.fi/, https://portals.broadinstitute.org/collaboration/giant/index.php/GIANT_consortium, http://www.nealelab.is/uk-biobank, https://gwas.mrcieu.ac.uk, http://magicinvestigators.org/downloads/.
