# Supplementary material for: Non-alcoholic fatty liver disease and gestational diabetes mellitus: a bidirectional two-sample mendelian randomization study
Source: BMC Endocr Disord. 2024 Mar 20;24:40. doi: 10.1186/s12902-024-01569-6 (PMC10953072; doi:10.1186/s12902-024-01569-6)
Supplement: Supplementary file 1 — Supplementary Material 1 [file 12902_2024_1569_MOESM1_ESM.pdf]

Non-alcoholic fatty liver disease and gestational diabetes mellitus : A bidirectional two-sample Mendelian randomization study

Supplementary online information		
**Content**		**Page**
**Table S1.**	Characteristics of instrumental variables of NAFLD and *F* statistics	2
**Table S2.**	Characteristics of instrumental variables of GDM and *F* statistics	2
**Table S3.**	Mendelian randomization results of the causal effects of potential shared influencing factors on NAFLD	3
**Table S4.**	Mendelian randomization results of the causal effects of potential shared influencing factors on GDM	4
**Figure S1**.	The result of leave-one-out sensitivity analysis of the causal effect of NAFLD on GDM	5
**Figure S2**.	The forest plot of the causal effect of NAFLD on GDM	5
**Figure S3**.	The scatter plot of the causal effect of NAFLD on GDM	6
**Figure S4**.	The result of leave-one-out sensitivity analysis of the causal effect of GDM on NAFLD	6
**Figure S5**.	The forest plot of the causal effect of GDM on NAFLD	7
**Figure S6**.	The scatter plot of the causal effect of GDM on NAFLD	7

**Table S1 Characteristics of instrumental variables of NAFLD and *F* statistics**							
**SNP**	**Effect allele**	**Other allele**	**EAF**	**Beta**	**SE**	***P***	***F***
rs17321515	G	A	0.476097	-0.15409	0.020931	1.81E-13	54.20
rs182611493	G	A	0.012616	0.453817	0.067899	2.33E-11	44.67
rs2642442	T	C	0.316965	0.13769	0.022383	7.67E-10	37.84
rs3747207	A	G	0.214703	0.369714	0.022649	6.74E-60	266.46
rs429358	C	T	0.156247	-0.19922	0.029761	2.17E-11	44.81
NAFLD, nonalcoholic fatty liver disease; SNP, single-nucleotide polymorphism; EAF, effect allele frequency; SE, standard error.							

**Table S2 Characteristics of instrumental variables of GDM and *F* statistics**							
**SNP**	**Effect allele**	**Other allele**	**EAF**	**Beta**	**SE**	***P***	***F***
rs34872471	C	T	0.202724	0.161767	0.017049	2.35E-21	90.03
rs7123869	C	T	0.020088	0.295913	0.045894	1.14E-10	41.57
rs7722200	C	T	0.31561	-0.14408	0.015255	3.56E-21	89.20
rs7756992	G	A	0.332859	0.102687	0.014676	2.62E-12	48.96
rs9275373	A	G	0.120313	0.158059	0.020838	3.32E-14	57.53
GDM, gestational diabetes mellitus; SNP, single-nucleotide polymorphism; EAF, effect allele frequency; SE, standard error.							

Table S3 Mendelian randomization results of the causal effects of potential shared influencing factors on NAFLD

Trait	SNPs	IVW		MR-Egger		WM		MR-PRESSO			$P_{pleiotropy}$	$P_{heterogeneity}$ (I^2)
		OR (95%CI)	*P*	OR (95%CI)	*P*	OR (95%CI)	*P*	SNPs	OR (95%CI)	*P*		
BMI	436	1.93 (1.52-2.44)	**<0.001**	2.26 (1.22-4.19)	**0.009**	2.06 (1.41-3.01)	**<0.001**	436	1.93 (1.52-2.44)	**<0.001**	0.580	<0.001 (19.61%)
WHR	27	2.87 (1.50-5.50)	**0.002**	9.93 (0.45-217.2)	0.157	3.28 (1.49-7.25)	**0.003**	27	2.87 (1.50-5.50)	**0.002**	0.427	0.061(31.57%)
TG	239	1.52 (1.25-1.86)	**<0.001**	1.30 (0.97-1.75)	0.081	1.47 (1.10-1.96)	**0.009**	239	1.52 (1.25-1.86)	**<0.001**	0.158	<0.001 (31.84%)
LDL-C	136	0.98 (0.77-1.25)	0.893	0.80 (0.57-1.14)	0.225	0.93 (0.66-1.32)	0.691	136	0.98 (0.77-1.25)	0.893	0.127	0.004 (26.16%)
HDL-C	272	0.70 (0.57-0.85)	**<0.001**	0.72 (0.53-0.97)	**0.032**	0.73 (0.52-1.04)	0.080	272	0.70 (0.57-0.85)	**<0.001**	0.805	0.007 (18.16%)
Apo A-1	230	0.71 (0.58-0.87)	**<0.001**	0.67 (0.49-0.93)	**0.017**	0.69 (0.50-0.96)	**0.027**	230	0.71 (0.58-0.87)	**<0.001**	0.670	0.002 (23.08%)
Apo B	151	1.03 (0.84-1.27)	0.764	0.81 (0.62-1.06)	0.132	0.79 (0.58-1.07)	0.131	151	1.03 (0.84-1.27)	0.764	**0.008**	<0.001 (29.15%)
HOMA-B	11	0.88 (0.32-2.43)	0.801	2.20 (0.07-67.5)	0.663	1.31 (0.33-5.24)	0.698	11	0.88 (0.32-2.43)	0.801	0.594	0.370 (7.71%)
HOMA-IR	6	1.67 (0.42-6.65)	0.467	5.81 (0.05-636.6)	0.504	0.87 (0.12-6.38)	0.895	6	1.67 (0.42-6.65)	0.467	0.613	0.440 (0.00%)
TV	98	2.34 (1.21-4.50)	**0.011**	1.40 (0.05-37.91)	0.843	2.44 (0.95-6.29)	0.065	98	2.34 (1.21-4.50)	**0.011**	0.756	0.300 (6.54%)
Computer	70	0.84 (0.41-1.70)	0.622	1.49 (0.03-74.3)	0.842	1.03 (0.37-2.88)	0.957	70	0.84 (0.41-1.70)	0.622	0.769	0.339 (5.86%)
Driving	6	2.87 (0.26-31.3)	0.388	0.00 (0.00-20804)	0.327	4.47 (0.22-89.0)	0.326	6	2.87 (0.26-31.3)	0.388	0.292	0.500 (0%)

Abbreviations: NAFLD, nonalcoholic fatty liver disease; SNPs, single-nucleotide polymorphisms; IVW, Inverse Variance Weighted; WM, Weighted median; MR- PRESSO, Mendelian randomization pleiotropy residual sum and outlier; OR, odds ratio; BMI, body mass index; WHR, waist-to-hip ratio; TG, triglycerides; LDL-C, low-density lipoprotein cholesterol; HDL-C, high-density lipoprotein cholesterol; Apo A-1, apolipoprotein A-I; Apo B, apolipoprotein B; HOMA-B, homeostasis model assessment of beta-cell function; HOMA-IR, homeostasis model assessment of insulin resistance; TV, Time spent watching television; Computer, Time spent using computer; Driving, Time spent driving.

Table S4 Mendelian randomization results of the causal effects of potential shared influencing factors on GDM

Trait	SNPs	IVW		MR-Egger		WM		MR-PRESSO			$P_{pleiotropy}$	$P_{heterogeneity}$ (I^2)
		**OR (95%CI)**	***P***	**OR (95%CI)**	***P***	**OR (95%CI)**	***P***	**SNPs**	**OR (95%CI)**	***P***		
BMI	430	1.63 (1.46-1.82)	**<0.001**	1.69 (1.26-2.25)	**<0.001**	1.74 (1.46-2.07)	**<0.001**	430	1.63 (1.46-1.82)	**<0.001**	0.806	<0.001 (31.86%)
WHR	24	1.64 (1.23-2.38)	**0.010**	0.38 (0.03-4.08)	0.431	1.37 (0.90-2.10)	0.139	24	1.64 (1.23-2.38)	**0.010**	0.234	0.001 (53.13%)
TG	238	1.27 (1.16-1.39)	**<0.001**	1.05 (0.93-1.19)	0.429	1.09 (0.97-1.24)	0.150	238	1.27 (1.16-1.39)	**<0.001**	<0.001	<0.001 (35.48%)
LDL-C	135	0.95 (0.85-1.06)	0.369	0.99 (0.84-1.16)	0.904	0.91 (0.78-1.07)	0.271	135	0.95 (0.85-1.06)	0.369	0.503	<0.001 (32.75%)
HDL-C	274	0.77 (0.71-0.84)	**<0.001**	0.84 (0.74-0.96)	**0.014**	0.83 (0.72-0.96)	**0.017**	274	0.77 (0.71-0.84)	**<0.001**	0.058	<0.001 (32.22%)
Apo A-1	232	0.82 (0.75-0.90)	**<0.001**	0.90 (0.78-1.03)	0.140	0.83 (0.72-0.95)	**0.008**	232	0.82 (0.75-0.90)	**<0.001**	0.127	<0.001 (31.14%)
Apo B	151	1.03 (0.94-1.13)	0.488	1.04 (0.92-1.18)	0.509	1.08 (0.95-1.23)	0.226	151	1.03 (0.94-1.13)	0.488	0.831	<0.001 (30.54%)
HOMA-B	5	0.57 (0.24-1.38)	0.212	0.47 (0.00-246.2)	0.827	0.61 (0.24-1.58)	0.310	5	0.57 (0.24-1.38)	0.212	0.953	0.198 (33.47%)
HOMA-IR	6	0.65 (0.36-1.17)	0.151	0.72 (0.11-4.88)	0.750	0.75 (0.34-1.65)	0.475	6	0.65 (0.36-1.17)	0.151	0.918	0.723 (0.00%)
TV	98	1.57 (1.15-2.15)	**0.005**	2.43 (0.50-11.74)	0.272	1.51 (1.01-2.26)	**0.045**	98	1.57 (1.15-2.15)	**0.005**	0.581	0.022 (23.61%)
Computer	70	0.75 (0.54-1.05)	0.096	0.87 (0.14-5.54)	0.884	0.67 (0.43-1.04)	0.077	70	0.75 (0.54-1.05)	0.096	0.875	0.062 (21.53%)
Driving	6	1.30 (0.16-10.7)	0.809	0.00 (0.00-2036)	0.299	1.39 (0.25-7.66)	0.703	6	1.30 (0.16-10.7)	0.809	0.287	<0.001 (76.14%)

Abbreviations: GDM, gestational diabetes mellitus; SNPs, single-nucleotide polymorphisms; IVW, Inverse Variance Weighted; WM, Weighted median; MR- PRESSO, Mendelian randomization pleiotropy residual sum and outlier; OR, odds ratio; BMI, body mass index; WHR, waist-to-hip ratio; TG, triglycerides; LDL-C, low-density lipoprotein cholesterol; HDL-C, high-density lipoprotein cholesterol; Apo A-1, apolipoprotein A-I; Apo B, apolipoprotein B; HOMA-B, homeostasis model assessment of beta-cell function; HOMA-IR, homeostasis model assessment of insulin resistance; TV, Time spent watching television; Computer, Time spent using computer; Driving, Time spent driving.

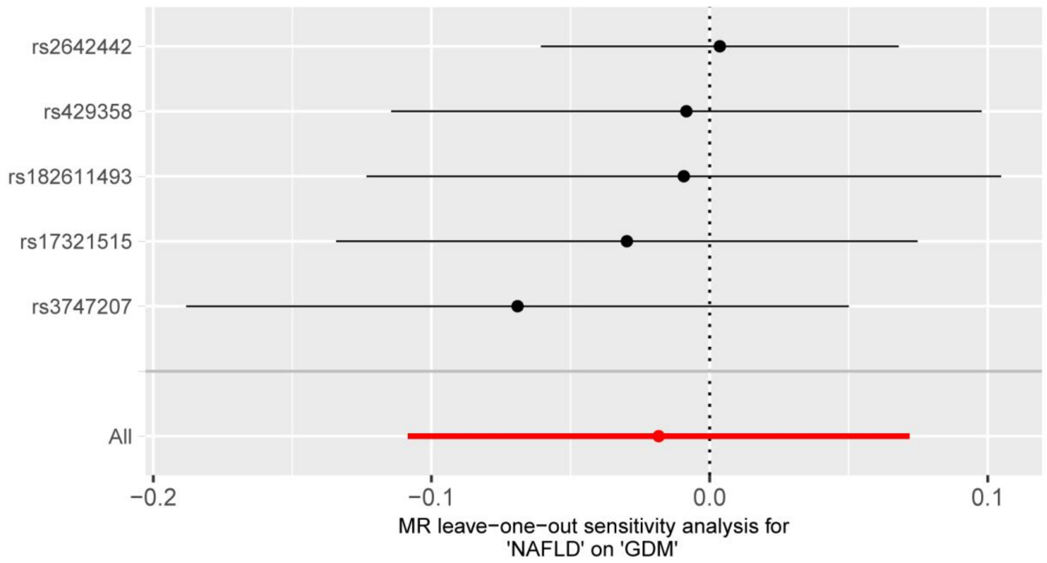

Fig S1. The result of leave-one-out sensitivity analysis of the causal effect of NAFLD on GDM

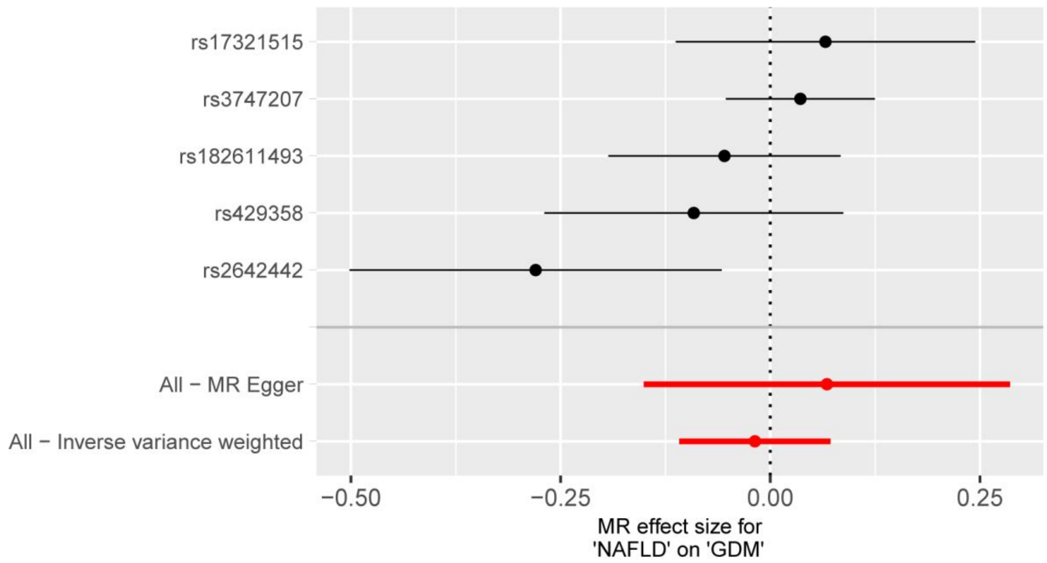

Fig S2. The forest plot of the causal effect of NAFLD on GDM

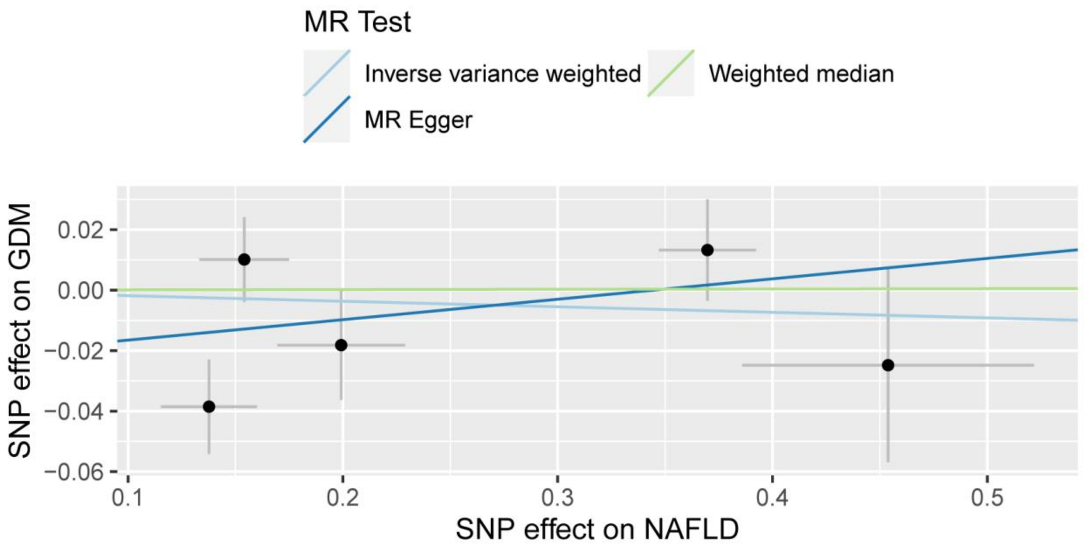

Fig S3. The scatter plot of the causal effect of NAFLD on GDM

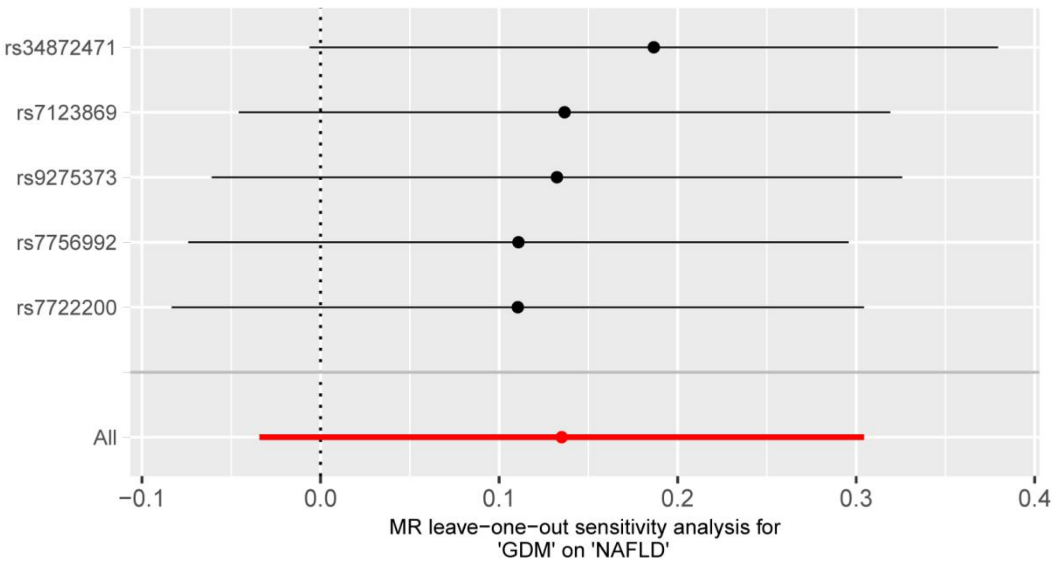

Fig S4. The result of leave-one-out sensitivity analysis of the causal effect of GDM on NAFLD

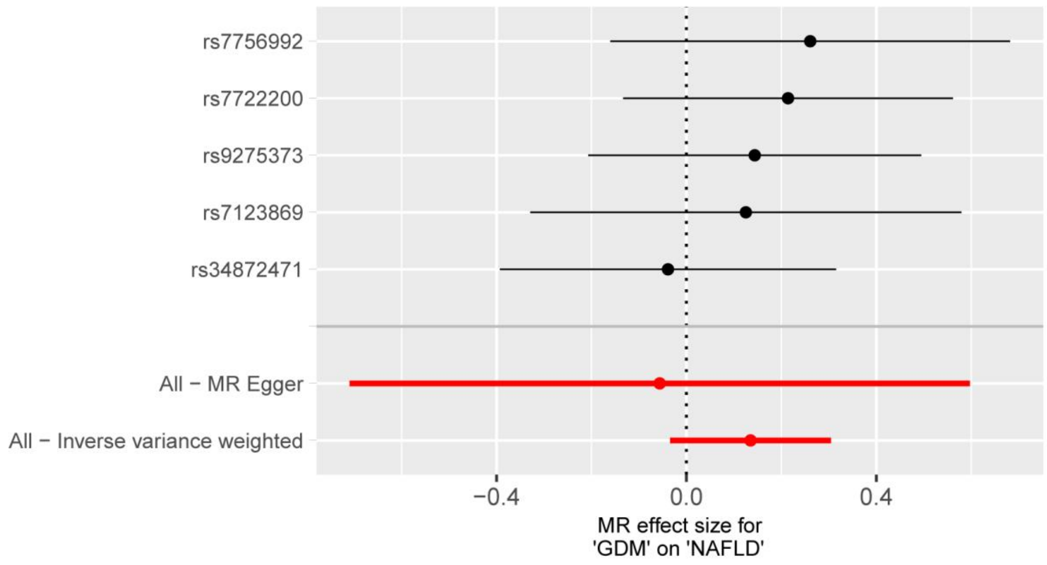

Fig S5. The forest plot of the causal effect of GDM on NAFLD

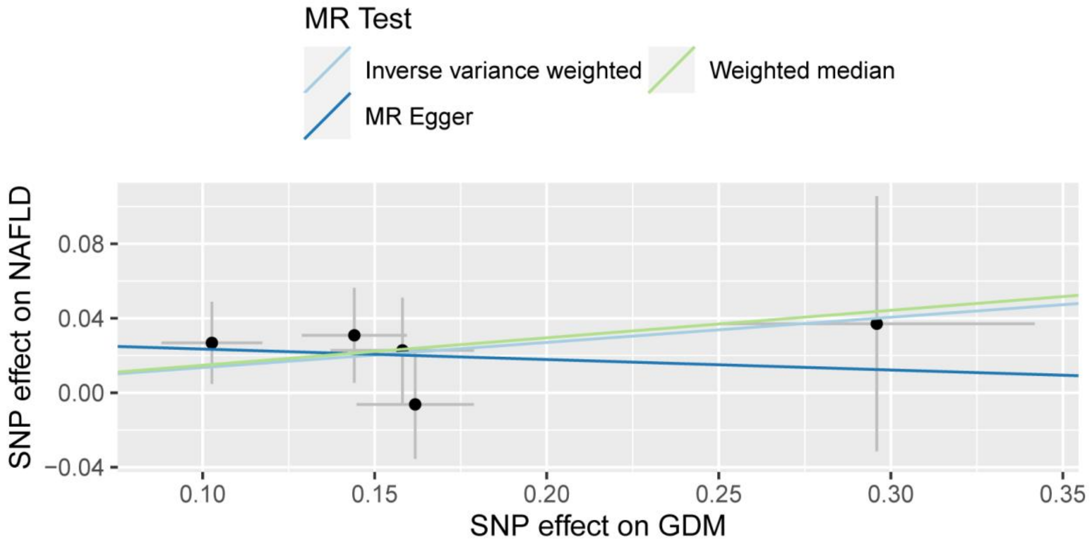

Fig S6. The scatter plot of the causal effect of GDM on NAFLD
